# Newborn weight change and childhood cardio-metabolic traits – a prospective cohort study

**DOI:** 10.1186/s12887-018-1184-x

**Published:** 2018-07-02

**Authors:** Maria João Fonseca, Milton Severo, Debbie A. Lawlor, Henrique Barros, Ana Cristina Santos

**Affiliations:** 1 EPIUnit - Instituto de Saúde Pública, Universidade do Porto, Rua das Taipas n° 135, 4050-600 Porto, Portugal; 2 Departamento de Ciências da Saúde Pública e Forenses e Educação Médica, Faculdade de Medicina, Universidade do Porto, Porto, Portugal; 3 Medical Research Council Integrative Epidemiology Unit at the University of Bristol, Bristol, UK; 4 School of Social and Community Medicine, University of Bristol, Bristol, UK

**Keywords:** Cardio-metabolic risk, Metabolic syndrome, Newborn weight loss

## Abstract

**Background:**

Newborn weight change (NWC) in the first 4 days represents short-term adaptations to external environment. It may be a key developmental period for future cardio-metabolic health, but this has not been explored. We aimed to determine the associations of NWC with childhood cardio-metabolic traits.

**Methods:**

As part of Generation XXI birth cohort, children were recruited in 2005/2006 at all public units providing obstetrical and neonatal care in Porto. Birthweight was abstracted from clinical records and postnatal anthropometry was obtained by trained examiners during hospital stay. NWC was calculated as ((minimum weight - birthweight)/birthweight) × 100. At age 4 and 7, children were measured and had a fasting blood sample collected. Fasting glucose, LDL-cholesterol, triglycerides, waist circumference, systolic and diastolic blood pressure were evaluated. This study included 312 children with detailed information on growth in very early life and subsequent cardio-metabolic measures. Path analysis was used to compute adjusted regression coefficients and 95% confidence intervals.

**Results:**

NWC was not associated with any cardio-metabolic traits at ages 4 or 7. Strong associations were observed between each cardio-metabolic trait at 4 with the same trait at 7 years. The strongest associations were found for waist circumference [0.725 (0.657; 0.793)] and LDL-cholesterol [0.655 (0.575; 0.735)].

**Conclusions:**

No evidence that NWC is related to childhood cardio-metabolic traits was found, suggesting that NWC should be faced in clinical practice as a short-term phenomenon, with no medium/long term consequences, at least in cardio-metabolic health. Our results show strong tracking correlations in cardio-metabolic traits during childhood.

**Electronic supplementary material:**

The online version of this article (10.1186/s12887-018-1184-x) contains supplementary material, which is available to authorized users.

## Background

Hypertension, central adiposity, high glucose levels and adverse lipid profile are damaging cardio-metabolic traits that co-occur in adults and children and are associated with future type 2 diabetes and coronary heart disease [1, 2]. There is evidence that exposures during early development play an important role in the future risk of this adverse cardio-metabolic health. Barker’s [3] initial description of an association between low birthweight and higher cardio-metabolic risk has been confirmed by other authors [4–7]. More recently, an association between higher birthweight and adverse cardio-metabolic health has also been reported, including in children [5, 8, 9]. Different patterns of postnatal weight change have also been found to associate with future cardio-metabolic health, and recent evidence suggests that body mass index (BMI) in childhood is more strongly related to adverse cardio-metabolic health than birthweight [10, 11].

These studies have not examined the association of weight change in the immediate postnatal period with later health outcomes, largely because few studies have such data. In the immediate postnatal period, newborns lose around 6% of their birthweight [12, 13]. Although this is considered a normal physiological process, there is considerable variation between newborns in the amount of weight lost during this period [12, 13]. Extreme values of newborn weight change (NWC) in the immediate postnatal period, which is mainly related to inadequate or excess hydration, are associated with adverse neonatal health [14, 15], but its association with subsequent cardio-metabolic health is unknown.

**Fig. 1:**
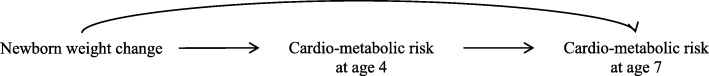
Hypothesized mechanism linking newborn weight change with cardio-metabolic risk at ages 4 and 7 years: an overall effect with direct and indirect components

We hypothesized that NWC during the first 96 h is associated with cardio-metabolic traits in later childhood, through developmental adaptations occurring during this period, when newborns have to rapidly adapt their energy intake and expenditure to external conditions [6]. Accordingly, our primary aim was to evaluate the association of NWC with cardio-metabolic traits assessed at age 4 and 7 years. We also explored whether the associations of NWC with these traits at age 7 were direct or mediated by the same traits at age 4 as represented in Fig. 1, and estimated all the paths represented in the figure. To our knowledge this is the first study to examine the associations of NWC with later cardio-metabolic traits.

## Methods

### Participants

The participants of this study are part of the Generation XXI birth cohort [16], assembled between April 2005 and August 2006, after delivery, during the hospital stay, at the five public units providing obstetrical and neonatal care in the metropolitan area of Porto, Portugal. Follow-up assessments of the cohort have been undertaken when the children were aged 4 years (April 2009 – July 2011), and 7 (April 2012 – January 2014).

**Fig. 2:**
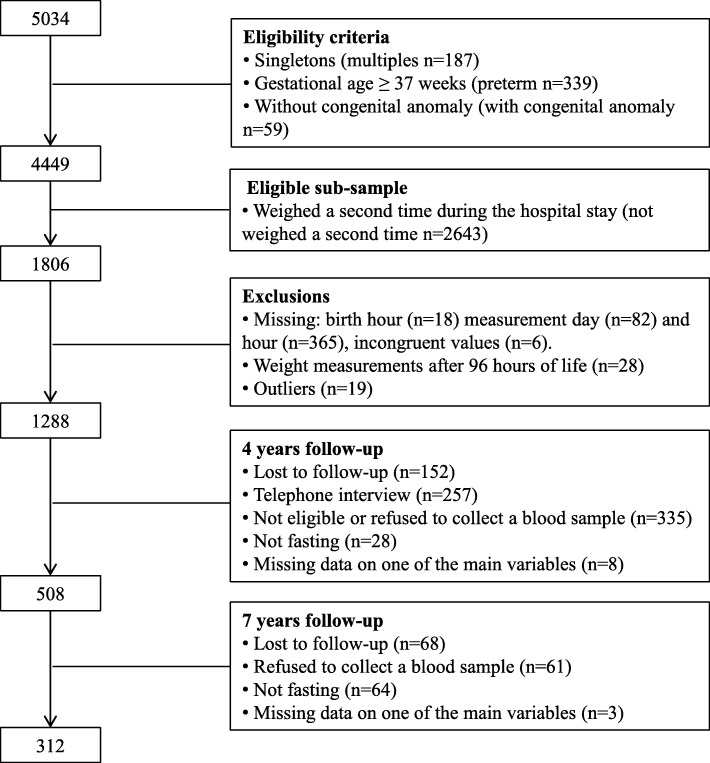
Study flow chart of participants

All newborns were routinely weighed at birth and, since November 2005, whenever possible, newborns additionally had a second weight measurement performed by a trained examiner during their hospital stay. Since November 2005, 5034 newborns were recruited to Generation XXI, of which 4449 were full-term singletons without known congenital anomalies. Of those 4449 children, a random sub-sample of 1806 newborns had the second neonatal weight measurement. This group of 1806 children were eligible for this study and of that 1806, 471 had missing information on exact time of measurement during hospital stay, 28 were measured after 96 h of life (the period of interest was the first 96 h of life) and 19 were considered outliers [1st/3rd quartile ±3 times the interquartile range corresponding to those with weight loss higher than 0.50% of birthweight per hour (*n* = 15) and those with weigh gain higher than 0.19% of birthweight per hour (*n* = 4)]. Of the remaining 1288, 312 children had complete data on all key variables and are the participants included in this study. Figure 2 shows the study flow chart of participants. Similar characteristics were found between participants and eligible non-participants (Additional file 1).

### Baseline evaluation

The second weight measurement of the newborn was performed by trained examiners, during the hospital stay, but independently of routine procedures. Newborns were weighted after the questionnaire to the mother, without clothes or diaper. The same digital scales were used (seca®) to weight all newborns to the nearest 1 g, after waiting for the scale to stabilize. The date and time of measurement were registered. The measurement time varied from 6.3 to 96.0 h of life, mean of 45.3 (SD 19.4) hours. We calculated NWC using the formula:

NWC(%) = ((estimated minimum weight – birthweight) / birthweight) × 100.

where estimated minimum weight was the predicted weight at 52.3 h of life - the mean *nadir* time of lowest weight in the first 96 h in European infants [13]. This was estimated for each child using a cubic regression model as described in the statistical analysis section.

Information on family and personal history of disease, socio-demographic characteristics, maternal pre-pregnancy anthropometric parameters, and intra-uterine exposures were collected during a face-to-face interview conducted during the hospital stay by trained interviewers. These interviews took place 24 to 72 h after delivery. Data on delivery and newborn characteristics, including birthweight and gestational age, were abstracted from clinical records by the same interviewers.

### Follow-up evaluations

At 4 and 7 years of age, trained researchers performed anthropometric and blood pressure measurements and obtained a fasting blood sample, according to standard procedures. Waist circumference measurements were taken with an inextensible tape measure to the nearest 0.1 cm, at the umbilicus level, with the child in a standing position, the abdomen relaxed, arms at the sides and feet positioned together [17]. Blood pressure was measured with an electronic sphygmomanometer (Omron®), with the child conformably sitting in a chair, with the cuff on the non-dominant arm, 2–3 cm above the elbow (without clothes compressing the arm). Two measurements of systolic (SBP) and diastolic (DPB) blood pressure, separated by at least 5 min, were taken after 10-min rest. If the difference between them was less than 5 mmHg for SBP or DBP, the mean was calculated. If the difference was larger than 5 mmHg a third measurement was taken and the mean of the 2 closest values was used [18]. After an overnight fast, a venous blood sample was collected before 11 a.m., according to standard procedures, after applying a topical analgesic cream (EMLA cream). This blood sample was centrifuged at 3500 rpm for 10 min and then the supernatant (serum) was stored at − 80 °C. Glucose was measured using UV enzymatic assay (hexokinase method), total and high density lipoprotein-cholesterol (for posterior calculation of LDL-C using Friedewald equation) [19], and triglycerides (TG) using an enzymatic colorimetric assay, in the Clinical Pathology Department of Centro Hospitalar São João, Porto, Portugal.

Our outcomes were: glucose, LDL-C, TG, waist circumference, SBP and DBP. In order to be able to compare magnitudes of association between different cardio-metabolic traits, we generated age- and sex-specific z-scores. For fasting glucose, LDL-C, TG and waist circumference this was done using the age- (in 6-months categories) and sex-specific means and standard deviations (SD) from the whole Generation XXI cohort. For SBP and DBP, we generated age-, sex- and height-specific z-scores using the means and standard deviations recommended by the American Academy of Pediatrics, in order to generate measures of BP that are independent of height (a major contribution to BP variation in children) [18]. High levels of the outcomes were considered when above the 90th percentile.

All the phases of the study complied with the Ethical Principles for Medical Research Involving Human Subjects expressed in the Declaration of Helsinki. The study was approved by the University of Porto Medical School/ Centro Hospitalar São João ethics committee and all parents or legal representative signed an informed consent according Helsinki.

STROBE checklist for the present manuscript can be found in Additional file 2.

### Statistical analysis

The estimated *nadir* time was 52.3 h of life, thus, weights used to calculate NWC were birthweight and the weight estimated at 52.3 h of life. A cubic model with random intercept and slope by subject [*weight*(*t*)~3241.442 + (−9.378) × *t* + 0.119 × *t*2 + (−0.0004) × *t*3 + *b*0i + *b*1i × *t*] was used to estimate the weight according to the newborn’s age represented as *t* in the formula (this analysis was performed using R version 2.14.1) [13].

Proportions were compared using the chi-square test, and means were compared using student’s t-test or ANOVA (analysis performed using SPSS version 21.0). Pearson correlations were also computed. Crude and adjusted linear regression coefficients (β) and 95% confidence intervals (95% CI) were computed using path analysis. Full information maximum likelihood estimation was used to handle missing values, assuming missing at random [20]. We conducted path analysis based on the theoretical model depicted in Fig. 1 and tested the fit of the model with potential confounders. The final model included NWC, maternal education, maternal pre-pregnancy BMI, gestational age, and birthweight as explanatory variables. Path analysis was performed with Mplus software (version 7); 95% confidence intervals were calculated by bootstrapping. The fit of the models was assessed using different indexes: the Comparative Fit Index (CFI) [21], the Tucker–Lewis Index (TLI) [22], and the Root Mean Square Error of Approximation (RMSEA) [23]. A good model fit is indicated by a CFI and TLI values ≥0.90 and values of RMSEA close to 0.

## Results

Table 1 shows the mean and standard deviation (or median and interquartile range for TG due to non-normal distribution) of NWC and all cardio-metabolic traits (glucose, LDL-C, TG, waist circumference, SBP and DBP) and also the number of children above the 90th percentile of the outcomes. Mean NWC was − 6.86% (ranging from − 15.03 and 5.30%). Mean values of all cardio-metabolic traits increased between 4 and 7 years, with the exception of LDL-C and TG which decreased.

**Table 1:** Characteristics of the study sample at baseline, 4 and 7 years follow-up

	Baseline	4 years follow-up	7 years follow-up
Newborn weight change (%), mean (SD)	−6.86 (2.32)	–	–
Glucose (mg/dL), mean (SD)	–	77.9 (7.9)	83.0 (5.7)
High glucose, n (%)	–	22 (7.1)	27 (8.7)
LDL-C (mg/dL), mean (SD)	–	107.0 (23.5)	99.8 (22.2)
High LDL-C, n (%)	–	30 (9.6)	31 (9.9)
Triglycerides (mg/dL), median (IQR)	–	61.0 (25.0)	55.0 (28.0)
High triglycerides, n (%)	–	30 (9.6)	28 (9.0)
Waist circumference (cm), mean (SD)	–	52.0 (4.2)	58.3 (6.2)
High waist circumference, n (%)	–	20 (6.4)	25 (8.0)
Systolic blood pressure (mmHg), mean (SD)	–	97.3 (7.6)	105.0 (8.7)
High systolic blood pressure, n (%)	–	23 (7.4)	66 (21.2)
Diastolic blood pressure (mmHg), mean (SD)	–	56.1 (7.8)	69.2 (7.4)
High diastolic blood pressure, n (%)	–	21 (6.7)	102 (32.7)

*Abbreviations*: *LDL-C* low density lipoprotein cholesterol, *SD* standard deviation

Table 2 presents unadjusted correlations of NWC and cardio-metabolic trait z-scores in childhood. There was no strong evidence of association of NWC with any of the cardio-metabolic traits at age 4 or 7. There were correlations between some cardio-metabolic traits at each age and traits at age 4 were positively associated with the same trait at age 7.

**Table 2:** Correlations of newborn weight change (in first 96 h) and cardio-metabolic risk factors z-scores in childhood

		4 years						7 years					
		Glucose	LDL-C	TG	Waist circumf.	SBP	DBP	Glucose	LDL-C	TG	Waist circumf.	SBP	DBP
	NWC	0.015	0.041	0.014	−0.015	0.071	0.057	0.025	0.007	0.046	0.044	0.108	−0.020
4 years	Glucose	1	0.039	0.036	0.151**	0.039	0.019	0.266***	0.081	0.049	0.090	0.096	0.062
	LDL-C	–	1	0.053	0.031	0.041	−0.027	0.015	0.667***	0.042	−0.032	− 0.044	− 0.007
	TG	–	–	1	0.053	0.047	0.086	−0.119*	0.064	0.295***	0.047	−0.043	−0.002
	Waist circumf.	–	–	–	1	0.226***	0.344***	0.091	−0.007	0.133*	0.776***	0.160**	0.097
	SBP	–	–	–	–	1	0.369***	0.033	−0.007	0.000	0.207***	0.367***	0.307***
	DBP	–	–	–	–	–	1	0.033	−0.028	−0.013	0.242***	0.164**	0.175**
7 years	Glucose	–	–	–	–	–	–	1	0.098	0.089	0.090	0.154**	0.116*
	LDL-C	–	–	–	–	–	–	–	1	0.120*	−0.020	0.047	0.078
	TG	–	–	–	–	–	–	–	–	1	0.135*	0.024	0.015
	Waist circumf.	–	–	–	–	–	–	–	–	–	1	0.262***	0.172**
	SBP	–	–	–	–	–	–	–	–	–	–	1	0.554***
	DBP	–	–	–	–	–	–	–	–	–	–	–	1

*Abbreviations*: *DBP* diastolic blood pressure, *LDL-C* low density lipoprotein cholesterol, *NWC* newborn weight change, *SBP* systolic blood pressure, *TG* triglycerides

*p < 0.05

**p < 0.01.

***p < 0.001.

Table 3 presents linear regression coefficients and 95% confidence intervals showing the adjusted total association of NWC with cardio-metabolic traits at ages 4 (model 1) and 7 (model 1) and the adjusted direct association of NWC with cardio-metabolic traits at age 7 (model 2), from the path analysis. These were consistent with the unadjusted association, with no strong evidence that NWC was associated with cardio-metabolic traits at age 4 or 7. Cardio-metabolic traits at age 4 were associated with the same trait at age 7, with the strongest associations observed for waist circumference [adjusted regression coefficient: 0.725 (0.657; 0.793)] and LDL-C [adjusted regression coefficient: 0.655 (0.575; 0.735)].

**Table 3:** Adjusted associations between newborn weight change, cardio-metabolic indicators at age 4 and cardio-metabolic indicators at age 7 from path analysis

	NWC (%)	Cardio-metabolic indicators at age 4 (z-score)
	β_1_ (95%CI)	β_5_ (95%CI)
Cardio-metabolic indicators at age 4		
Model 1		
Glucose z-score	0.004 (−0.043; 0.051)	–
LDL-cholesterol z-score	0.017 (−0.031; 0.065)	–
Triglycerides z-score	0.004 (−0.040; 0.049)	–
Waist circumference z-score	−0.003 (− 0.046; 0.039)	–
Systolic blood pressure z-score	0.017 (−0.016; 0.049)	–
Diastolic blood pressure z-score	0.015 (−0.018; 0.048)	–
Cardio-metabolic indicators at age 7		
Model 1		
Glucose z-score	0.008 (−0.035; 0.051)	–
LDL-cholesterol z-score	0.001 (−0.045; 0.047)	–
Triglycerides z-score	0.017 (−0.025; 0.060)	–
Waist circumference z-score	0.019 (−0.022; 0.060)	–
Systolic blood pressure z-score	0.031 (−0.006; 0.067)	–
Diastolic blood pressure z-score	−0.010 (− 0.040; 0.020)	–
Model 2		
Glucose z-score	0.007 (−0.035; 0.048)	0.235 (0.136; 0.334)*
LDL-cholesterol z-score	−0.012 (− 0.045; 0.022)	0.655 (0.575; 0.735)*
Triglycerides z-score	0.016 (−0.024; 0.057)	0.281 (0.180; 0.383)*
Waist circumference z-score	0.021 (−0.008; 0.025)	0.725 (0.657; 0.793)*
Systolic blood pressure z-score	0.025 (−0.009; 0.058)	0.369 (0.251; 0.488)*
Diastolic blood pressure z-score	−0.013 (− 0.042; 0.016)	0.153 (0.050; 0.256)*

*Abbreviations*: *NWC* newborn weight change, *LDL* low density lipoprotein, *β* regression coefficient, *CI* confidence interval

Cardio-metabolic indicator at age 4 (Model 1) ≈ β_0_ + β_1_ (NWC) + β_2_ (maternal education) + β_3_ (maternal pre-pregnancy BMI) + β_4_ (gestational age) + β_5_ (birth weight)

Cardio-metabolic indicator at age 7 (Model 1) ≈ β_0_ + β_1_ (NWC) + β_2_ (maternal education) + β_3_ (maternal pre-pregnancy BMI) + β_4_ (birth weight)

Cardio-metabolic indicator at age 7 (Model 2) ≈ β_0_ + β_1_ (NWC) + β_2_ (maternal education) + β_3_ (maternal pre-pregnancy BMI) + β_4_ (birth weight) + β5 (same cardio-metabolic indicator at 4)

*p < 0.05.

## Discussion

We evaluated the association of NWC during the first 96 h of life with childhood cardio-metabolic outcomes. To our knowledge, no previous studies have longitudinally examined these associations. Similar to previous reports, we found that newborns lost on average 7% of their birthweight in the first 96 h of life. No robust evidence that NWC influenced childhood cardio-metabolic traits was found. Tracking correlation coefficients between ages 4 and 7 years were found for all cardio-metabolic traits, with the strongest being for waist circumference and LDL-C.

The prevalence of adverse levels of cardio-metabolic traits, such as central obesity, impaired glucose tolerance, dyslipidemia, and hypertension, has been increasing among children [1, 8]. Also, the co-occurrence of adverse levels of these cardio-metabolic traits, known as metabolic syndrome, has recently been identified in children, and its prevalence is around 3% but tending to increase [1, 24]. The tracking found in regards to all cardio-metabolic traits indicates that prevention should start as early as possible, because, according to our results, a 4-year-old child with adverse levels of cardio-metabolic traits will probably be a 7-year-old child also with adverse levels, and, according to previous studies, will probably have adverse levels across life [25]. Bearing in mind this remark, early screening of the levels of cardio-metabolic traits in children may indeed be justified from the point of view of cardio-metabolic chronic disease prevention. So, studies showing the normal cardio-metabolic traits distribution during childhood are of importance [26–28].

The biological mechanisms by which weight change during critical periods could lead to chronic diseases remain unclear. Theories of early programming hypothesize that under- or over- nutrition and other insults, when occurring during critical periods of development, may lead to permanent alterations in tissues’ structure and functions, and in the neuroendocrine systems [6, 29]. Nevertheless, the exact timing of programming that contributes to the medium/long-term risk continues to be debated. There is some evidence that different early growth patterns may precede the development of adverse levels of cardio-metabolic traits later in life [5, 30–33], with a recent genetic study in 22,769 Europeans finding genetic evidence for a causal link between age and BMI at adiposity rebound and subsequent cardio-metabolic ill-health. However, the medium/long-term consequences of weight changes during the very first days of life had not been studied until now because very few studies have a second weight measure after birth within the first 96 h.

The nearest studies we could find to this very early period of rapid weight loss and major adaptation, were two recent studies, one of which examined weight change in the first week of life [34] and the second the first two weeks [35]; both found higher weight gain in these periods was associated with greater odds of overweight in adulthood and childhood, respectively. However, these studies considered a period when the NWC *nadir* had already occurred, so all newborns evaluated in those studies were already gaining weight. On the other hand, in our time frame, almost all newborns lost weight, because it focused in the first 96 h of life. Additionally, none of the studies examine NWC in relation to the cardio-metabolic traits analyzed in the present study.

Extreme values of NWC in the immediate postnatal period are associated with adverse health outcomes in the neonatal period, such as hypernatremic dehydration, which can cause serious medical complications, such as disseminated intravascular coagulation, cerebrovascular accidents and even death, or, on the other hand, over hydration and related morbidities such as bronchopulmonary dysplasia, intraventricular–periventricular hemorrhage, necrotizing enterocolitis and patent ductus arteriosus [14, 15]. Even though extreme values of NWC has adverse effects in the neonatal period, it does not appear to have long term adverse cardio-metabolic effects.

### Limitations and strengths

A large proportion of the main cohort participants were not included in our analyses. However, distributions of maternal and neonatal characteristics between participants and eligible non-participants were similar. Our finding that NWC during the first 96 h was not associated with childhood cardio-metabolic traits might be due to lack of statistical power, but the point estimates were all close to the null and the 95%CI were narrow, suggesting that we had adequate power to obtain precise estimates and rule out an important association.

As weight measurements were not taken at regular periods for each newborn, it is possible that the precise *nadir* of NWC was not detected. Nevertheless, a systematic review [12] found a mean NWC ranged from − 5.7% to − 6.6% and a *nadir* around the 2nd and 3rd days following birth. These results support our methodology as we found a mean NWC of − 6.7% occurring at 52.3 h of life [13], suggesting that we correctly estimate the actual *nadir* in our sample.

We used path analysis to explore associations. This is an extension of regression analysis, which allows for simultaneous estimation of the interrelations between variables. We used it here because it is a useful method for comparing the magnitudes of effects between variables with complex interrelations or to test the plausibility of mediation effects [36].

Our exposure – NWC – measures the growth occurring within 96 h, which is a very narrow period. It is known that the measurement error on weight change is higher and the capacity to identify associations between growth periods and outcomes reduced, when the measurements are closer in time and the weight change is smaller [37]. Since measurement error in NWC is unlikely to be related to later child cardio-metabolic outcomes (the midwives and trained researchers who measured birth and later weight would have no way of knowing the future cardio-metabolic traits of the newborns they were assessing), the expectation would be for this random error to attenuate the observed associations, however the bias would also depend on relationships of the error to other explanatory variables in the model. We doubt it would fully explain the consistent null results we find across all traits.

Although cardio-metabolic traits in early childhood track throughout childhood (as we showed) and into adulthood, clinically abnormal values of cardio-metabolic risk factors, such as fasting glucose or SBP, are unusual in childhood [38], and it is possible that the medium/long-term effects of NWC on cardio-metabolic health are not yet fully evident at the ages we have assessed. Future research conducted as this population ages will enable this possibility to be explored.

## Conclusions

We found no strong evidence that NWC is related to cardio-metabolic traits at age 4 or 7, suggesting that NWC does not influence the development of adverse cardio-metabolic outcomes at least up to age 7. Thus, NWC should be faced in clinical practice as a short-term phenomenon, with no medium/long term consequences, at least in cardio-metabolic health.

## Additional files

Additional file 1: Comparison between participants and eligible non participants regarding maternal, pregnancy, delivery and newborn characteristics. Table with the comparison between participants and eligible non participants. (DOCX 35 kb)

Additional file 2: STROBE Statement—Checklist of items that should be included in reports of cohort studies. A STROBE checklist for the present manuscript. (DOC 85 kb)

## Abbreviations

BMI: Body mass index
DBP: Diastolic blood pressure
LDL-C: Low density lipoprotein cholesterol
NWC: Newborn weight change
SBP: Systolic blood pressure
TG: Triglycerides
