# Supplementary material for: Newborn weight change and childhood cardio-metabolic traits – a prospective cohort study
Source: BMC Pediatr. 2018 Jul 2;18:211. doi: 10.1186/s12887-018-1184-x (PMC6029387; doi:10.1186/s12887-018-1184-x)
Supplement: Supplementary file 1 — Comparison between participants and eligible non participants regarding maternal, pregnancy, delivery and newborn characteristics. Table with the comparison between participants and eligible non participants. (DOCX 35 kb) [file 12887_2018_1184_MOESM1_ESM.docx]

| Additional file 1. Comparison between participants and eligible non participants regarding maternal, pregnancy, delivery and newborn characteristics. | | | | |
| --- | --- | --- | --- | --- |
|  |  | Eligible non participants n=1494 | Participants n=312 | p |
| **Maternal characteristics** | |  |  |  |
|  | Education (years), mean (SD) | 10.7 (4.2) | 11.3 (4.1) | 0.038 |
|  | Age at delivery (years), mean (SD) | 28.6 (5.8) | 29.7 (5.1) | 0.002 |
|  | Pre-pregnancy BMI (kg/m^2^), mean (SD) | 24.0 (4.4) | 24.2 (4.1) | 0.555 |
| **Pregnancy characteristics** | |  |  |  |
|  | Tobacco smoke during 3^rd^ trimester, n (%) | 211 (14.5) | 43 (14.0) | 0.826 |
|  | Weight gain (kg), mean (SD) | 13.5 (5.6) | 13.4 (5.5) | 0.877 |
| **Delivery characteristics** | |  |  |  |
|  | Caesarean delivery, n (%) | 461 (31.0) | 93 (30.0) | 0.717 |
| **Newborn characteristics** | |  |  |  |
|  | Gestational age (weeks), mean (SD) | 39.0 (1.1) | 39.1 (1.1) | 0.948 |
|  | Sex, n (%) |  |  |  |
|  | Male | 748 (50.1) | 165 (52.9) |  |
|  | Female | 746 (49.9) | 147 (47.1) | 0.365 |
|  | Birth weight (g), mean (SD) | 3249 (427) | 3282 (404) | 0.214 |
|  | Newborn weight change, mean (SD) | -6.70 (2.33) | -6.86 (2.32) | 0.272 |
| Abbreviations: BMI, body mass index; SD, standard deviation. | | | | |
